# Urinary human polyomavirus and papillomavirus infection and bladder cancer risk

**DOI:** 10.1038/bjc.2011.519

**Published:** 2011-11-24

**Authors:** J Polesel, T Gheit, R Talamini, N Shahzad, O Lenardon, B Sylla, C La Vecchia, D Serraino, M Tommasino, S Franceschi

**Affiliations:** 1 Unit of Epidemiology and Biostatistics, IRCCS Centro di Riferimento Oncologico, 33081 Aviano (PN), Italy; 2 International Agency for Research on Cancer, 150 Cours Albert Thomas, 69372 Lyon Cedex 08, France; 3 Unit of Urology, Azienda Ospedaliera Santa Maria degli Angeli, 33170 Pordenone (PN), Italy; 4 Department of Epidemiology, The Mario Negri Institute for Pharmacological Research, 20156 Milan, Italy; 5 Department of Occupational Medicine, Università degli Studi di Milano, 20133 Milan, Italy

**Keywords:** human polyomavirus, human papillomavirus, bladder cancer

## Abstract

**Background:**

The association of transitional cell carcinomas of the bladder (TCB) with *Schistosoma haematobium* suggested a possible role of infections in the aetiology of TCB.

**Methods:**

In all, 114 TCB cases and 140 hospital controls from Pordenone Province were enrolled within an Italian multi-centric case–control study. Urine samples were screened for DNA from five human polyomaviruses (HPyV) (JCV, BKV, MCV, WUV, and KIV); SV40; and 22 mucosal human papillomaviruses (HPV) using highly sensitive PCR assays. Odds ratios (ORs) and corresponding confidence intervals (CIs) were computed for risk of TCB by HPyV- or HPV-positivity using unconditional logistic regression.

**Results:**

Human polyomavirus prevalence was similar in TCB cases (71.7%) and controls (77.7%) (OR for TCB=0.85; 95% CI: 0.45–1.61). JCV was the most frequently detected HPyV type. No individual HPyV showed a significant association. Among cases, HPyV-positivity was not associated with tumour characteristics, but it was significantly lower in women than men and among current and former smokers than never smokers. Human papillomavirus was detected in seven cases and five controls (OR=1.52; 95% CI: 0.42–5.45).

**Conclusion:**

The present small study does not support an involvement of HPyV or HPV infection in TCB aetiology in immunocompetent individuals. Differences in HPyV-positivity by sex and smoking may derive from differences in either acquisition or persistence of the infection.

Incidence rates of bladder cancer in Italian men are among the highest worldwide (Ferlay *et al*, 2010). Tobacco smoking and exposure to aromatic amines, ionising radiation, and cyclophosphamide are well-established risk factors for bladder cancer (Silverman *et al*, 2006). The associations with *Schistosoma haematobium* (Bedwani *et al*, 1998) and kidney transplantation (Vajdic *et al*, 2006) and the possible association with recurrent urinary tract infections (La Vecchia *et al*, 1991) raised interest in a possible role of infectious agents, including the mucosal human papillomavirus (HPV) types that are responsible for the vast majority of cancers of the anogenital tract (IARC, 2007), and, more recently, human polyomaviruses (HPyV) (Barbanti-Brodano *et al*, 2006; Rollison *et al*, 2007; Roberts *et al*, 2008).

Five HPyV have been known for some time (i.e., JCV, BKV, MCV, WUV, and KIV) and four additional types have been recently characterised (Johne *et al*, 2011; Scuda *et al*, 2011). BKV has been most intensively studied in relation to bladder cancer as its major sites of persistence are the cells of the kidney and urinary tract (Jiang *et al*, 2009) and it induces nephropathy in renal transplant patients. Both BKV and JCV were detected in the urine of a substantial proportion of immunosuppressed and immunocompetent individuals (Jiang *et al*, 2009). Most previous work on HPyV (Geetha *et al*, 2002; Fioriti *et al*, 2003; Weinreb *et al*, 2006) or HPV (IARC, 2007) and bladder cancer consisted, however, of small-sized case series that did not include any comparison group or relied on antibody seroprevalence only (Newton *et al*, 2005).

To further evaluate the possible role of viral infections in bladder cancer, we carried out the first case–control study on the relationship between bladder cancer risk and the presence of HPyV in urine samples. The presence of HPV DNA in bladder cancer cases and controls was also compared.

## Materials And Methods

### Study subjects

The present report deals with the first cases of transitional cell carcinoma of the bladder (TCB) and control subjects consecutively enrolled in the province of Pordenone between August 2004 and July 2007 within the framework of an on-going multi-centric case–control study of TCB.

Cases (*n*=124) were patients above 18 years of age with incident histologically or cytologically confirmed TCB admitted to major general hospitals in the study area. Controls (*n*=151) were patients admitted for a wide spectrum of acute conditions to the same hospitals where cases had been interviewed. Subjects admitted for diseases related to tobacco smoking or alcohol consumption, or any disorder that might have induced long-term modification of diet were excluded from the control group. The reasons for hospital admission among controls were traumatic orthopaedic disorders (36.4%); other orthopaedic disorders (28.6%); acute surgical conditions (27.9%); and a range of other illnesses, including nose, ear, skin, or dental disorders (7.1%). Refusal rate was below 5% in either cases and controls. Transplant recipients were ineligible as cases or controls and there was no indication of severe immunosuppression in the medical records of any of the study subjects. All study subjects signed an informed consent, according to the recommendations of the Board of Ethics of the study hospitals.

### Questionnaire and biological samples

Trained interviewers administered a structured questionnaire to cases and controls during their hospital stay. The questionnaire collected information on socio-demographic factors, lifestyle habits, diet, a problem-oriented medical history, family history of cancer, occupational history, exposure to selected carcinogens, and hair dye use. Detailed information on lifetime history of tobacco smoking and alcohol drinking was elicited and validated (D’Avanzo *et al*, 1996).

We collected a 50-ml sample of first-voided urine before study subjects had undergone any treatment. Standard clean-catch procedures for urine collection were applied to prevent sample contamination. Half of the sample (25 ml) was stored at −80°C and the remaining 25 ml were put in CytoLyt solution and stored at 4°C.

### DNA extraction

Urine samples in CytoLyt solution were concentrated using the Amicon Ultra-15 centrifugal filters (Millipore Corp., Bedford, MA, USA). In all, 15 ml of urine was added to the filter unit for a centrifugation at 4000 **g** for 15 min. After centrifugation, DNA extraction was performed using the MagNA Pure Compact robot (Roche Diagnostics GmbH, Mannheim, Germany) with the nucleic acid isolation kit I – large volume according to the manufacturer's instructions. To monitor the possible occurrence of cross-contamination between the different specimens during DNA extraction, tubes containing water only were also included and blindly processed in the following steps of the study. The DNA quality of samples was checked by amplifying a fragment of the *β*-globin gene using Hot Start PCR, performed according to the standard protocols. All analyses were carried out under conditions of pre- and post-PCR separation.

### Polyomavirus typing

#### Multiplex PCR conditions

First, urine samples were screened using a multiplex PCR-based assay that identified the presence of any of five HPyV (JCV, BKV, MCV, WUV, and KIV) and one simian PyV (SV40). Six pairs of specific primers were designed in order to amplify a fragment of the Large T Ag gene of 253 bp for MCV and 256 bp for the other HPyV and SV40 (sequences available upon request). The accession numbers of the GenBank sequences that we used as references, with the corresponding HPyV given in parentheses, were NC_009539 (WUV), EF520287 (KIV), NC_001699 (JCV), NC_001538 (BKV), EU375804 (MCV), and NC_001669 (SV40). Oligonucleotides were synthesised by MWG Biotech (Ebersberg, Germany).

Forty amplification cycles were run in a GeneAmp PCR System 2700 (Applied Biosystems, Weiterstadt, Germany) with a 94°C denaturation step for 30 s, a 63°C annealing step for 3 min, and a 72°C extension step for 1.30 min, including an initial denaturation step of 15 min at 95°C and a final extension step of 10 min at 72°C. The 25-*μ*l reactions comprised 1 × multiplex PCR Kit buffer (Qiagen, Hilden, Germany), containing 3 mM MgCl_2_, dNTP mix and HotStart*Taq* DNA polymerase, 0.2 *μ*mol l^–1^ of each primer, and 10 *μ*l of DNA. In all, 5 *μ*l of the PCR products was checked by ethidium bromide agarose gel electrophoresis.

In order to evaluate the sensitivity of our assay, a multiplex PCR was performed using serial dilutions of DNA (from 1000 to 0 copies of viral genome) from HPyV types and SV40 as template. A PCR was positive down to 10 copies of viral genome. The presence of a large number of primers in the multiplex PCR, therefore, did not hamper the sensitivity of the assay. In order to evaluate specificity, human genomic DNA was mixed with individual HPyV genomes and tested in the multiplex PCR. The test was positive when HPyV DNA was included, while it was always negative when human genomic DNA only was used as template (data not shown). In addition, the specificity of present findings was further strengthened by direct DNA sequencing of HPyV PCR fragments.

#### Sequencing

In order to identify individual HPyV types and SV40, all samples that were positive by multiplex PCR were sequenced. After enzymatic purification with 0.4 *μ*l of Exonuclease I (10 U ml^–1^) (New England Biolabs, Hertfordshire, UK) and 0.2 *μ*l of Shrimp Alkaline Phosphatase (1 U ml^–1^) (USB Corp., Cleveland, OH, USA) at 37°C for 15 min and an inactivation step at 80°C for 15 min, the PCR products were sequenced by the fluorescent dye dideoxy termination method using an ABI Prism 377 DNA sequencer (PE Applied Biosystems, Foster City, CA, USA). For the sequencing reaction, the same primers were used as for the PCR reaction.

### HPV typing

The detection of HPV DNA was performed using a highly sensitive and specific assay recently developed in our laboratory using a multiplex PCR combined to the Luminex suspension array technology (Luminex Corp., Austin, TX, USA). This assay is able to detect 19 mucosal high-risk and potential high-risk HPV types (16, 18, 26, 31, 33, 35, 39, 45, 51, 52, 53, 56, 58, 59, 66, 68a, 70, 73, and 82) (Gheit *et al*, 2006; Schmitt *et al*, 2010). Another type of HPV68 (68b) and two mucosal low-risk HPV types (HPV6 and HPV11) were recently added to the assay.

### Statistical analysis

The present analyses were restricted, according to the virus under evaluation, to (1) *β*-globin-positive subjects (96 cases and 96 controls); and (2) *β*-globin-negative subjects who were positive for HPyV DNA (17 cases and 43 controls) or HPV DNA (one case and four controls). For HPyV analyses, 113 cases (median age: 67 years; range: 37–79 years) and 139 controls (median age: 67 years; range: 36–78 years) were considered. Conversely, 97 cases (median age: 68 years; range: 37–79 years) and 100 controls were included in HPV analyses (median age: 68 years; range: 36–78 years). Odds ratios (ORs) for TCB and for HPyV-positivity or HPV-positivity and their corresponding 95% confidence intervals (CIs) were calculated by means of unconditional logistic regression models (Breslow and Day, 1980), adjusted for sex, age, education, alcohol drinking, and smoking habits as reported in Tables 1, 2, 3, 4.

## Results

Transitional cell carcinoma of the bladder cases and controls reported similar education level (Table 1). Smoking was strongly associated with TCB risk (OR for current *vs* never smoking=9.15; 95% CI: 3.92–21.37), with a significant risk trend for number of cigarettes (*P*<0.01), while no significant association was found with alcohol consumption.

Human polyomavirus-positivity in urine was similar in cases (71.7%) and controls (77.7%) (OR=0.85; 95% CI: 0.45–1.61) (Table 2). JCV was the most frequently detected HPyV (65.5% and 70.5% in cases and controls, respectively), and it was associated with an OR of 1.19 (95% CI: 0.62–2.28). Positivity for BKV and MCV was rarer but not significantly different in TCB cases and controls. Corresponding CIs were, however, broad. WUV, KIV, and SV40 were never detected. Mucosal HPV DNA was detected in seven cases (including HPV31, 35, 45, 58, 70 and HPV56 in two cases) and five controls (including HPV33, 51, 58 and HPV56 in two controls) (OR=1.52; 95% CI: 0.42–5.45) (Table 2).

Table 3 shows age, histological type, grade, and stage among TCB cases by HPyV-positivity. The majority of TCB cancers in our study showed papillary features and were detected at stage 0 (zero). No significant differences in tumour characteristics were found between HPyV-positive and HPyV-negative cases.

Factors associated with HPyV-positivity are shown in the combination of cases and controls (Table 4) since no relationship had emerged between HPyV-positivity and case/control status. Human polyomavirus-positivity did not vary by age group, education level, and drinking habit. Conversely, HPyV-positivity was lower in women than men (OR=0.29; 95% CI: 0.14–0.57) and among current (OR=0.32; 95% CI: 0.13–0.79) and former (OR=0.34; 95% CI: 0.13–0.90) smokers than never smokers. The risk of HPyV-positivity was also inversely related to the number of siblings (OR for 0–1 *vs* ⩾6 siblings=2.82; 95% CI: 1.07–7.41; *P* for trend=0.03) (Table 4). All associations with HPyV-positivity were confirmed when ORs were additionally adjusted for tobacco smoking, as appropriate, and when analyses were restricted to TCB cases or controls, and men or women (data not shown).

## Discussion

Our present case–control study confirms that DNA from HPyV, notably JCV, BKV, and MCV, is frequently detectable in the urine of immunocompetent individuals, but it is found in similar proportions in TCB cases and controls. Human papillomavirus DNA was seldom detected but, as for HPyV, its presence was not significantly associated to TCB risk. No difference in HPyV-positivity among TCB cases was found by age group, histological type, tumour grade, or stage.

Seroprevalence of antibodies against BKV and JCV in adults was reported to be, respectively, over 90% and 50% (Knowles, 2006). After primary infection in childhood or adolescence, BKV and JCV are known to persist in urothelial cells of the kidney, ureter, and bladder although it is unclear whether these viruses enter a latent state or maintain a low level of viral gene expression and replication at these sites (Jiang *et al*, 2009). Intermittent replication is supported by periodic viral excretion in the urine. Our findings agree with previous reports of JCV and BKV viruria of 20–60% and 5%, respectively, among immunocompetent individuals (Knowles, 2006).

An involvement of HPyV in bladder cancer aetiology had been suggested by a few case series that showed high prevalence of various markers of HPyV, mainly BKV, in TCB (Fioriti *et al*, 2003; Weinreb *et al*, 2006; Abend *et al*, 2009; Maginnis and Atwood, 2009). Human polyomaviruses can transform rodent and human cells and are carcinogenic in animal models (Barbanti-Brodano *et al*, 2006). In addition, they encode a tumour (T) antigen protein complex that targets the product of several tumour suppressor genes (Moore and Chang, 2010). Although they showed a significant difference between cases and controls, Weinreb *et al* (2006), in a previous investigation on urine samples, reported prevalences of BKV (9.8% among TCB cases and 3.1% among controls) similar to those reported in our study (6.2% and 3.6%, respectively). Rollison *et al* (2007) reported that BKV DNA was found in 5.4% of 70 patients with TCB but T-antigen expression was not demonstrated by immunohistochemistry in BKV DNA-positive cancer tissue. Roberts *et al* (2008) used immunohistochemistry to assess the prevalence of T-antigen from any HPyV in TCB. None of the 20 consecutive non-renal transplant patients was positive for T-antigen. Out of eight TCBs in renal transplant patients, one showed a strong nuclear staining with T-antigen, in agreement with another single-case report from a renal transplant patient (Geetha *et al*, 2002). In a case–control study nested in a cohort, there was no significant difference in seroprevalence or titre of antibodies against BKV between 9 TCB cases and 45 controls (Newton *et al*, 2005). Very little is known on TCB and HPyV other than BKV and there is no clear evidence of an association of HPyV with any human cancer except for Merkel cell tumour (Moore and Chang, 2010).

JCV and BKV are believed to be transmitted early in life through respiratory or urino-oral mode but risk factors for acquisition or reactivation of the infection, other than immunosuppression, have never been reported (Jiang *et al*, 2009). In our study, HPyV-positivity did not vary by age group but it was significantly less frequent in women than men and in current or former smokers than never smokers. It was also inversely correlated to family size although not to education level. The reasons for the lower frequency of HPyV-positivity in women and smokers and former smokers are unclear but our present findings were not modified by inclusion of TCB cases and controls or controls only or stratification by smoking or sex. As we relied on HPyV DNA presence in urine, rather than serum anti-HPyV antibodies, the risk factors that we identified might be associated with acquisition, persistence, or reactivation of HPyV infection.

No association between HPV-positivity and TCB was found in our study. Notably, no HPV16 or 18, the types that greatly predominate in known HPV-related cancers (IARC, 2007), were detected among TCB cases. A large number of case series have been published on the presence of mucosal HPV types in TCB (IARC, 2007; Cai *et al*, 2011; Shigehara *et al*, 2011; Yavuzer *et al*, 2011). The prevalence of HPV DNA varied widely (from 0% to 80%) in PCR-based studies (IARC, 2007). A case–control study showed a significantly higher prevalence of HPV DNA in the urine of 78 TCB cases (46%) compared with 59 controls (14%) who had undergone transurethral resection of the prostate for benign prostatic hyperplasia (Cai *et al*, 2011). Contamination from the lower genital tract during the acquisition of urine or exfoliated cells from the bladder is, however, of special concern in these studies on account of the frequent presence of HPV infection in the lower genital tract (IARC, 2007).

Urine samples have limited value to evaluate the presence of HPV infection in the external male genitalia as the yield of *β*-globin and HPV DNA is worse than in exfoliated cell samples from the glans, corona, prepuce, and shaft of the penis (Dunne *et al*, 2006). The aim of our study was, however, to assess the presence of HPV infection in the bladder and, therefore, sampling of exfoliated cells from the male external genitalia would have been clearly inappropriate. On the other hand, it was impossible to use more invasive sampling procedures (e.g., bladder tissue biopsies), as they would have been impossible to obtain from the control group. The restriction of our present study to *β*-globin-positive urine samples should have at least in part obviated sample quality problems.

Unique strengths of our present study include the availability of a well-comparable control group and of a wide range of information on lifestyle risk factors for TCB. In addition, our study was the first one to rely on highly sensitive and specific PCR assays that allowed the detection of DNA of five HPyV and SV40 and all the most common mucosal HPV types. An important weakness of our present study is the limited number of subjects involved. Confidence intervals around our ORs were, therefore, broad and did not allow to rule out the presence of positive association, especially with BKV and HPV. Another major weakness was lack of further assessment of viral DNA presence or immunohistochemistry findings in cancer tissue samples.

In conclusion, our small study does not support an involvement of HPyV or HPV infection in the aetiology of TCB in immunocompetent individuals. Lower detection of HPyV DNA in women and smokers might be related to differences in either acquisition or persistence and reactivation of the infection.

## Figures and Tables

**Table 1:** Distribution of 114 bladder cancer cases and 140 hospital controls according to socio-demographic characteristics and selected variables

	**Cases**	**Controls**	
	***n* (%)**	***n* (%)**	**OR (95% CI)^a^**
*Sex*			
Men	95 (83.3)	114 (81.4)	
Women	19 (16.7)	26 (18.6)	
			
*Age (years)*			
<65	42 (36.8)	50 (35.7)	
65–74	43 (37.7)	61 (43.6)	
⩾75	29 (25.4)	29 (20.7)	
			
*Education (years)*			
<7	54 (47.4)	71 (50.7)	Ref.
7–11	40 (35.1)	44 (31.4)	1.27 (0.68–2.38)
⩾12	20 (17.5)	25 (17.9)	1.12 (0.50–2.49)
*χ*^2^ for trend			0.19; *P*=0.66
			
*Smoking habit*			
Never	15 (13.2)	45 (32.1)	Ref.
Former	54 (47.4)	77 (55.0)	2.25 (1.10–4.60)
Current	45 (39.5)	18 (12.9)	9.15 (3.92–21.37)
Intensity (cig per day)^b^			
<15	13 (11.4)	8 (5.7)	4.92 (1.64–14.73)
⩾15	32 (28.1)	10 (7.1)	11.23 (4.00–31.53)
*χ*^2^ for trend			22.08; *P*<0.01
			
*Drinking habit (drinks per week)*			
<21	43 (37.7)	65 (46.4)	Ref.
⩾21	71 (62.3)	75 (53.6)	1.60 (0.87–2.95)

Abbreviations: CI=confidence interval; OR=odds ratio.

a Estimated from unconditional logistic regression, adjusted for sex, age, education, and smoking habit, as appropriate.

b Among current smokers only.

**Table 2:** Odds ratios (ORs) of bladder cancer and corresponding 95% confidence intervals (CIs) by prevalence of DNA from human polyomaviruses (HPyV), simian virus 40 (SV40), or human papillomavirus (HPV) in the urine

		**Positive**		
	**Negative^a^**	**Cases**	**Controls**	
	**Ca:Co**	***n* (%)**	***n* (%)**	**OR (95% CI)^b^**
HPyV	32:31	81 (71.7)	108 (77.7)	0.85 (0.45–1.61)
				
*HPyV type* c				
JCV	39:41	74 (65.5)	98 (70.5)	1.19 (0.62–2.28)
BKV	106:134	7 (6.2)	5 (3.6)	1.45 (0.32–6.55)
MCV	110:131	3 (2.7)	8 (5.8)	0.51 (0.12–2.13)
WUV	113:139	0 (0.0)	0 (0.0)	—
KIV	113:139	0 (0.0)	0 (0.0)	—
				
SV40	113:139	0 (0.0)	0 (0.0)	—
				
HPV	90:95	7 (7.2)^d^	5 (5.0)^e^	1.52 (0.42–5.45)

a Reference category.

b Estimated from unconditional logistic regression and adjusted for sex, age, education, tobacco smoking, and alcohol drinking.

c Including multiple infection with JCV–BKV in one case, and multiple infection with JCV–MCV in two cases and three controls.

d Including HPV31, 35, 45, 58, 70 and HPV56 in two subjects.

e Including HPV33, 51, 58 and HPV56 in two subjects.

**Table 3:** Detection of human polyomavirus (HPyV) DNA in the urine according to age and tumour characteristics among 113 bladder cancer cases

	**HPyV DNA**		
	**Positive**	**Negative**	
	***n* (%)**	***n* (%)**	**Fisher test**
*Age (years)*			*P*=0.39
<65	27 (65.9)	14 (34.1)	
65–74	34 (79.1)	9 (20.9)	
⩾75	20 (69.0)	9 (31.0)	
*χ*^2^ for trend			
			
*Histological type*			*P*=1.00
Transitional cell carcinoma	18 (72.0)	7 (28.0)	
Papillary transitional cell carcinoma	63 (71.6)	25 (28.4)	
			
*Grade*			*P*=1.00
Well differentiated	11 (73.3)	4 (26.7)	
Moderately differentiated	28 (71.8)	11 (28.2)	
Poorly differentiated/undifferentiated	40 (71.4)	16 (28.6)	
Unknown	2 (66.7)	1 (33.3)	
			
*Stage*			*P*=0.50
0	50 (74.6)	17 (25.4)	
I	15 (75.0)	5 (25.0)	
II or worse	15 (62.5)	9 (37.5)	
Unknown	1 (50.0)	1 (50.0)	

**Table 4:** Odds ratios (ORs) for the presence of human polyomavirus (HPyV) DNA in urine and corresponding 95% confidence intervals (CIs) according to selected variables among 252 study subjects

	**HPyV DNA**		
	**Positive**	**Negative**	
	***n* (%)**	***n* (%)**	**OR (95% CI)^a^**
*Age (years)*			
<65	69 (76.7)	21 (23.3)	1^b^
65–74	79 (76.0)	25 (24.0)	0.93 (0.47–1.84)
⩾75	41 (70.7)	17 (29.3)	1.00 (0.45–2.21)
*χ*^2^ for trend			0.00; *P*=0.97
			
*Sex*			
Men	165 (79.7)	42 (20.3)	1^b^
Women	24 (53.3)	21 (46.7)	0.29 (0.14–0.57)
			
*Education (years)*			
<7	92 (73.6)	33 (26.4)	1^b^
7–11	65 (77.4)	19 (22.6)	1.16 (0.58–2.35)
⩾12	32 (74.4)	11 (25.6)	0.94 (0.39–2.26)
*χ*^2^ for trend			0.00; *P*=1.00
			
*Smoking habit*			
Never	50 (83.3)	10 (16.7)	1^b^
Former	95 (73.6)	34 (26.4)	0.34 (0.13–0.90)
Current	44 (69.8)	19 (30.2)	0.32 (0.13–0.79)
			
*Drinking habit (drinks/week)*			
<21	75 (70.0)	32 (30.0)	1^b^
⩾21	114 (78.1)	31 (21.9)	1.07 (0.55–2.06)
			
*Number of siblings* c			
⩾6	26 (61.9)	16 (38.1)	1^b^
4–5	45 (75.0)	15 (25.0)	1.65 (0.68–4.01)
2–3	70 (76.9)	21 (23.1)	2.29 (0.97–5.40)
0–1	47 (81.0)	11 (19.0)	2.82 (1.07–7.41)
*χ*^2^ for trend			4.92; *P*=0.03

a Estimated from unconditional logistic regression and adjusted for case-control status, sex, age, as appropriate.

b Reference category.

c Sum does not add up to the total because of one missing value.
